# *N*-alkylimidazolium Salts Functionalized with *p*-Coumaric and Cinnamic Acid: A Study of Their Antimicrobial and Antibiofilm Effects

**DOI:** 10.3390/molecules24193484

**Published:** 2019-09-26

**Authors:** Oscar Forero-Doria, Ramiro Araya-Maturana, Anggela Barrientos-Retamal, Luis Morales-Quintana, Luis Guzmán

**Affiliations:** 1 Instituto de Química de Recursos Naturales, Universidad de Talca, P.O. Box 747, Talca 3460000, Chile; oforero@utalca.cl (O.F.-D.); raraya@utalca.cl (R.A.-M.); anggela.br@gmail.com (A.B.-R.); 2 Programa de Investigación Asociativa en Cáncer Gástrico (PIA-CG), Universidad de Talca, Talca 3460000, Chile; 3 Multidisciplinary Agroindustry Research Laboratory, Instituto de Ciencias Biomédicas, Universidad Autónoma de Chile, Talca 3460000, Chile; luis.morales@uautonoma.cl; 4 Departamento de Bioquímica Clínica e Inmunohematología, Facultad de Ciencias de la Salud, Universidad de Talca, P.O. Box 747, Talca 3460000, Chile

**Keywords:** Antibiofilm effects, antimicrobial agents, cinnamoylimidazole salts, ionic liquids

## Abstract

The bacterial resistance to antibiotics has compromised the therapies used for bacterial infections. Nowadays, many strategies are being carried out to address this problem. Among them, the use of natural compounds like cinnamic and *p*-coumaric acids stands out. Nevertheless, their utilization is limited because of their unfavorable physicochemical properties. Due to the lack of new therapeutic alternatives for bacterial infections, novel strategies have emerged, such as the use of ionic liquids; given that they can show a broad spectrum of antibacterial activity, this is why we herein report the antibacterial and antibiofilm activity of a series of *N*-alkylimidazolium salts functionalized with *p*-coumaric and cinnamic acids. The results from this study showed better antibacterial activity against Gram-positive bacteria, with a predominance of the salts derived from coumaric acid and a correlation with the chain length. Additionally, a lower efficacy was observed in the inhibition of biofilm formation, highlighting the antibiofilm activity against *Staphylococcus aureus*, which decreased the production of the biofilm by 52% over the control. In conclusion, we suggest that the salts derived from *p*-coumaric acid are good alternatives as antibacterial compounds. Meanwhile, the salt derived from cinnamic acid could be a good alternative as an antibiofilm compound.

## 1. Introduction

Many applications and innovations have been reported in the field of ionic liquids (ILs), which are salts composed of ions that exist in the liquid state at room temperature [1]. Normally, they are formed by organic cations that are linked with an organic or inorganic anion [2]. Most cations have an aromatic structure with nitrogen atoms in the aromatic ring, nitrogen heterocycles, while the anions usually show a higher diversity [3].

ILs have been used in different areas with multiple applications for both production and technological processes; for example, in chemistry, fundamental advances have been obtained by synthetic chemistry, producing an electro-colloidal interface; in biology, ILs have found use in enzymatic and cell biocatalysis, for protein stabilization and biodiesel production; and in physics and technology, ILs have been used in batteries and solar panels [4]. In recent years, the use of ILs as drug-releasing agents and in combination with pro-drugs has opened up new possibilities in the treatment of diseases [5,6].

It has been shown that ILs exhibit antibacterial activity, which may be attributable to one or both ions, giving rise to double-acting antimicrobial ILs [6]; however, the mechanism of action by which they act as antimicrobials is still unknown. IL solutions share structural and mechanical analogies with established biocides and cationic surfactants, such as quaternary ammonium compounds [7,8]. The common characteristics of a hydrophilic head group with one or more hydrophobic “tails” observed in both ILs and cationic surfactants (benzalkonium chloride, chloride/cetylpyridinium bromide, or cetrimonium bromide), suggest a common mechanism of action, which would be alteration of the integrity of the cell membrane. These similarities extend towards a tendency for ILs to aggregate in solution, forming micelles and presenting surface tension, observed when increasing the lipophilicity [7,9,10], commonly tuned through the alkyl chain substituent [11].

The occurrence of multiple resistance to penicillin, methicillin, and other agents has compromised the therapies used for bacterial infections [12]. The pathogens in which there has been an increase in the resistance to antibiotics are *Staphylococcus aureus*, *Enterococcus* spp., *Pseudomonas aeruginosa*, *Escherichia coli*, and *Klebsiella* spp. [13]. Additionally, these bacteria can form a biofilm, which is an aggregate of organized microorganisms that live inside an extracellular polymeric matrix produced by them, colonizing an inert surface or a living tissue [14]. This is a prokaryotic survival strategy, which gives the bacteria significant advantages, since it provides protection against environmental fluctuations of humidity, temperature, and pH, as well as protecting them against the efficacy of antibiotics [15].

Different strategies have been carried out to face the continuous increase in the resistance to antibiotics; one of these is the use of naturally occurring antimicrobial compounds, among which the use of cinnamic and coumaric acid stands out. Cinnamic acid is a natural phenolic compound present in various plant sources [16]. *p*-coumaric acid can be present as a free acid or in an esterified form in many vegetables, fruits, and graminaceous plants [17]. Both compounds have shown antibacterial activity against different bacterial strains [18,19,20]. Nevertheless, despite these activities, their utilization is limited because of their unfavorable physicochemical properties, especially their very poor water-solubility and low redox stability [21,22].

Given that ILs can show a broad spectrum of activity, affecting Gram-positive and -negative bacteria, the aim of this work was to determine the antibacterial and antibiofilm activity of *N*-alkylimidazolium salts functionalized with *p*-coumaric and cinnamic acid, and to evaluate the minimum inhibitory concentration (MIC) of these derivatives with different chain lengths and their action on the formation of a biofilm in pathogenic bacteria.

## 2. Results and Discussion

### 2.1. Chemistry

**Scheme 1:**
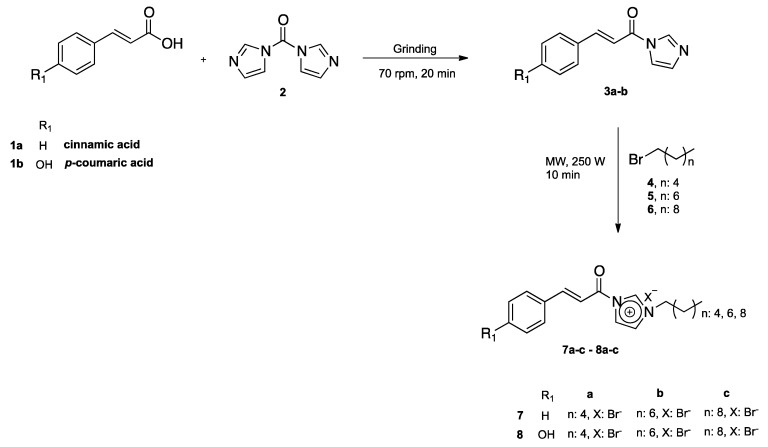
Synthesis of imidazolium salts derived from hydrocinammic-imidazol derivatives **7a**–**c** and **8a**–**c**.

**Scheme 2:**
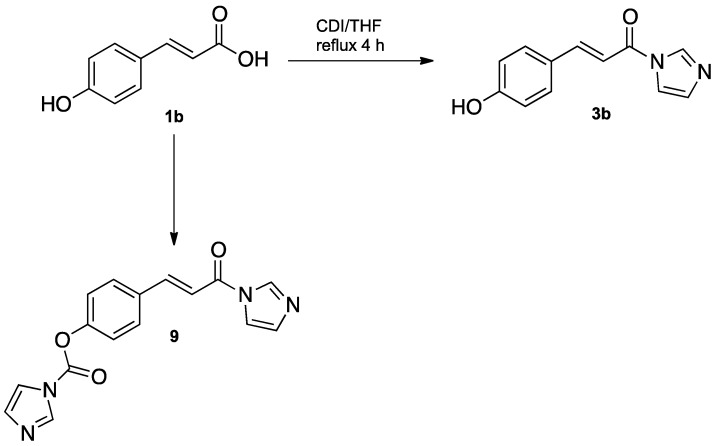
Synthesis in the condensed phase of *p*-coumaric-imidazol derivative **3b**.

The synthesis of *N*-cinnamoyl salts (**7a**–**c** and **8a**–**c**) is shown in Scheme 1. The first step for the synthesis of *N*-cinnamoyl salts was the production of amides **3a**–**b**. In an attempt to obtain **3a**–**b**, we tested the reaction of 1,1-carbonyldiimidazole (CDI) (**2**) with *p*-coumaric acid **1b** in a 1:1 equivalent molar ratio (Scheme 2). Briefly, the reaction mixture, with THF as the solvent and under nitrogen atmosphere, was refluxed (4 h) and its progress was monitored by TLC. The TLC plates showed multiple spots in the reaction mixture; the GC-MS analysis of the reaction crude evidenced the formation of **3b** and **9** with 40% and 30% conversion, respectively.

One of the reasons for the low yield presented in the formation of amide **3b** was the formation of by-product **9** due to the nucleophilic nature of the -OH phenolic present in the *p*-coumaric acid **1b**. Cappelli et al. [23] reported the formation of this by-product in the construction of hyaluronic acid derivatives. The researchers used 2 eq of CDI for the construction of **3b**, resulting in the formation of the latter together with **9** (Scheme 2).

Due to the low yield obtained in the formation of **3b** and the formation of by-product (**9**), the search for an efficient methodology for the construction of these amides was imperative. Verma et al., 2012 [24], reported the synthesis of a series of amides using CDI in solvent-free conditions. This methodology consists of macerating the corresponding acid with the CDI to obtain different acyl imidazoles; key intermediates in obtaining amides without the use of any dry organic solvent and in inert atmosphere for an average time of 5–10 min. Based on the experimental evidence previously described and motivated by the use of green approaches in our synthetic plan, we used this mechanochemical synthesis for the construction of amides **3a**–**b** (Scheme 1). The grinding reaction of the hydroxycinnamic acids **1a**–**b** with CDI was carried out in a 1:1 equivalent ratio, in a mortar grinder (FRITSCH: Pulverisette 2) for 20 min, which produced a viscous yellow oil for the cinnamic acid **1b**, and the derivative obtained from cinnamic acid **1a** was a colorless oil; the reactions were monitored by TLC until total consumption of the reagents was reached. Subsequently, the resulting amides were purified by column chromatography (CC) using a DCM/MeOH 95:5 elution mixture. The obtained amides **3a** (74%) and **3b** (92%) showed excellent yields, without the formation of by-products. It is important to highlight the regioselectivity in the amidation of **1b**, since the amide was obtained on the acid group and the formation of the carbamate **9** was not observed. In this sense, Patil et al. (2008) described the regioselective 6-*O*-tritylation/silylation (etherification) of methyl *α*-d-glucopyranoside or their derivatives under solvent-free conditions by planetary ball milling (dry grinding) [25]. Finally, the reaction of **3a** and **3b** with alkyl bromides (hexyl, octyl, and decyl chain lengths) was carried out in microwave and solvent-free conditions, as was described in a previous work [26].

The end of the reaction was the obtention of the salts **7a**–**c**, marked by the separation of colorless viscous oil; toluene was decanted to remove any unreacted starting materials and solvents, allowing this phase to be isolated. Subsequently, the viscous oil was rinsed with diethyl ether (4 × 3 mL), and the latter layer was then separated by decantation and placed in a high vacuum for 4 h to obtain the salts **7a**–**c**. On the other hand, the formation of the salts **8a**–**c** took place adequately. Chromatographic purification was carried out using Hexane/EtOAc 7:3 and the salts were obtained as creamy solids. The yields and experimental considerations are discussed in the experimental part of this paper.

### 2.2. Determination of the Minimum Inhibitory Concentration (MIC) by Microdilution in a 96-Well Plate

**Figure 1:**
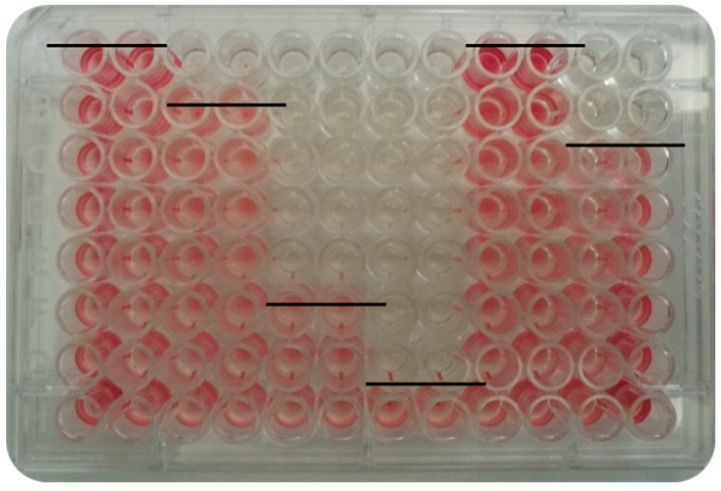
Minimum inhibitory concentration (MIC) determination by microdilution in a 96-well plate. The line indicates the last dilution where bacterial viability was not evidenced. Columns (1–12): different *N*-alkylimidazolium salts (in duplicate). Rows (A–H): seriated dilutions of each salt (2 - 0.015626 mM).

The MIC value was reported as the last dilution in which no bacterial viability was observed, using the redox indicator 2,3,5-triphenyltetrazolium chloride (Figure 1).

None of the precursors studied (**3a** and **3b**), at the concentrations used, showed antibacterial activity (Table 1). In Gram-positive bacteria (*S. aureus* and *S. epidermidis*), the salts derived from cinnamic acid showed a correlation between the antibacterial activity and the length of the alkyl chain, with a lower MIC being obtained as the alkyl chain increased. By adding a hydroxyl group to the molecule (coumaric acid), an improvement in the antibacterial activity was seen in the salts that had an alkyl chain of 8 and 10 carbons (four and two times, respectively) in comparison with those that did not possess the modification and had the same length of the chain; however, no difference in the antibacterial activity was seen when the alkyl chain had six carbons.

In Gram-negative bacteria, minimal antibacterial activity was observed against *P. aeruginosa*, with an MIC value of over 2 mM in both cases. In the case of the antibacterial activity against *Acinetobacter baumannii*, the same MIC was observed in the salts with six carbons; nevertheless, a better effect was observed in the salts with a chain length of 8 and 10 carbons without a hydroxyl group, contrary to what was observed in Gram-positive bacteria.

Since there is a great variety of etiological agents that cause bacterial infections and the emergence of multiple resistance to antibiotics, where the main ones would be the Gram-positive bacteria (*S. aureus*, *S. pyogenes*, and *Staphylococcus* coagulase negative), followed by the Gram-negative bacteria (Enterobacteria and non-fermenting bacilli) [27], correct identification of the pathogenic species, in addition to the study of antibiotic susceptibility, becomes essential. Additionally, biofilm production of the bacteria involved must be considered, since it is estimated that 65% of bacterial infections are associated with a bacterial biofilm [28].

As seen in other studies, imidazole-based ILs have shown good antimicrobial activity. Docherty and Kulpa, 2005, tested the toxicity and antimicrobial activity of ILs derived from imidazole, which exhibited significant antimicrobial activity in pure cultures of *E. coli*, *S. aureus*, *B. subtilis*, *P. fluorescens*, and *S. cerevisiae* [29]. Wei, et al., 2012, evaluated the antimicrobial activity of 23 cinnamoyl derivatives against *B. subtilis*, *E. coli*, and *S. cerevisiae*, showing that most of them display good antimicrobial activity against these microorganisms, especially against *S. cerevisiae* [30].

In this work, it was evidenced that the alkyl chain in the structure is mandatory to obtaining antibacterial activity at the concentrations used. The salts with chain lengths of 6, 8, or 10 carbons of the alkyl substituent, showed antibacterial activity dependent of the length of the chain, which indicates that the alkyl group significantly improves the antibacterial activity of ILs, as Luczak et al., 2010, also showed in 1-alkyl-3-methylimidazole derivatives in which the antibacterial activity improved when adding an alkyl chain [9].

Regarding the MIC values of the *N*-cinnamoyl salts synthesized, a resistance to most of them was evidenced in *P. aeruginosa* at the concentration used. The same results were obtained by Santos et al., 2014, who tested nine ILs containing an imidazole, colinium, or phosphonium cation, and showed that *P. aeruginosa* was the most tolerant bacterium to imidazole-based IL, being the only microorganism studied that grew in the presence of these ILs [31]. In *S. aureus*, *S. epidermidis*, and *A. baumannii*, it was found that an increase in the length of the chain increased the antibacterial activity, with the 10-carbon chain being the largest used in this work. In this respect, Garcia et al., 2014, evaluated the antimicrobial activity of ILs with long chains (from 6 to 14 carbon atoms), with an amide functional group and a cation of methylimidazole or pyridinium, against Gram-positive and Gram-negative bacteria and fungi, showing that the ILs with more than 8 carbon atoms in the alkyl chain had a broad spectrum of action [32]. This is consistent with the results obtained, since the salts with the longest chain (**7c** and **8c**) presented the best antibacterial activity. 

The antibacterial activity (measured as the MIC value) of the 10-carbon IL (**7c**) was 32 times better than the salt with the alkyl chain of 6 carbons (**7a**) for *S. aureus*, 64 times better for *S. epidermidis*, and 8 times better for *A. baumannii* (Table 1). Venkata et al., 2012, evaluated the antibacterial activity of 1-alkyl-3-methylimidazole, which had better antibacterial activity against Gram-positive bacteria (*S. aureus*) compared to Gram-negative bacteria (*P. aeruginosa*) [33], consistent with the results obtained in this work. The salts that had a hydroxyl group in their structure (*p*-coumaric derivative) improved the antibacterial activity against Gram-positive bacteria, decreasing the MIC value up to four times compared with the salts with no hydroxyl group. For example, salt **8b** presented a better activity against *S. aureus* and *S. epidermidis*. On the contrary, in Gram-negative bacteria, the addition of the hydroxyl group decreased the antibacterial activity, showing higher MIC values (up to four times higher), as in the case of **8c** in *A. baumannii*. This could be due to differences in the cell wall of Gram-positive and Gram-negative bacteria, so further studies should be carried out to determine the cause of these differences with the addition of a hydroxyl group.

Due to the best correlation between the antibacterial activity and the length of the chain being observed in the molecules with an eight-carbon chain length (**7b** and **8b**), these were used to test the antibiofilm activity against *Staphylococcus aureus*, *Staphylococcus epidermidis*, and *Acinetobacter baumannii*. Because of the high resistance presented by *P. aeruginosa* to the salts at the concentration used, the antibiofilm activity in this bacterium was not tested.

### 2.3. Determination of Antibiofilm Activity in 96-well Plates

The antibiofilm activity was calculated by the formation of a biofilm, comparing the absorbances measured in the wells that had the salt with the absorbance measured in the positive control, which was considered 100% biofilm formation (Table 2).

In *S. aureus*, inhibitory activity of the biofilm was evidenced by over 50% (CMI × 2) of the salt not having a hydroxyl group, which was statistically higher than the salt with the hydroxyl group, while minor activity was found in MIC × 1/2, with no differences among them. On the other hand, no differences were observed between the salts at a concentration of MIC × 2 in *S. epidermidis*; however, a total loss of activity was found in **7b** at a concentration of MIC × 1/2, and a similar situation was found in *A. baumannii*. Nevertheless, at a concentration of CMI × 2, the salt **7b** presented a percentage of inhibition of the biofilm statistically higher than that of **8b**.

The results showed a lower efficacy compared with the antibacterial activity, since the formation of a biofilm at the concentration used (MIC × 1/2) was not completely inhibited. Ferrer et al., 2017, analyzed the effect of ten antibiotics on the biofilm formation of clinical isolates of *S. aureus* and *S. epidermidis*, showing that, in some of the antibiotics, using sub-inhibitory concentrations, the formation of a biofilm was even stimulated [34]. In **8b**, no significant variation in biofilm formation was observed in any of the bacteria, for both concentrations used. Nevertheless, when **7b** was used at a concentration equivalent to twice the MIC, an inhibition in the formation of biofilm was observed, being higher in *S. aureus*, decreasing the production of biofilm by 52%. Venkata et al., 2012, evaluated the antibiofilm activity of 1-dodecyl-3-methylimidazole in *S. aureus* and *P. aeruginosa*, and obtained better antibiofilm activity against Gram-positive bacteria than Gram-negative [33], which is consistent with our results, where it was observed that the antibiofilm activity was better in *S. aureus*. More studies on the antibiofilm activity of these salts are required, since, due to the absence of previous reports of ILs equal to those used in this investigation, there is no point of comparison.

### 2.4. Molecular Dynamic (MD) Simulations Analysis

**Figure 2:**
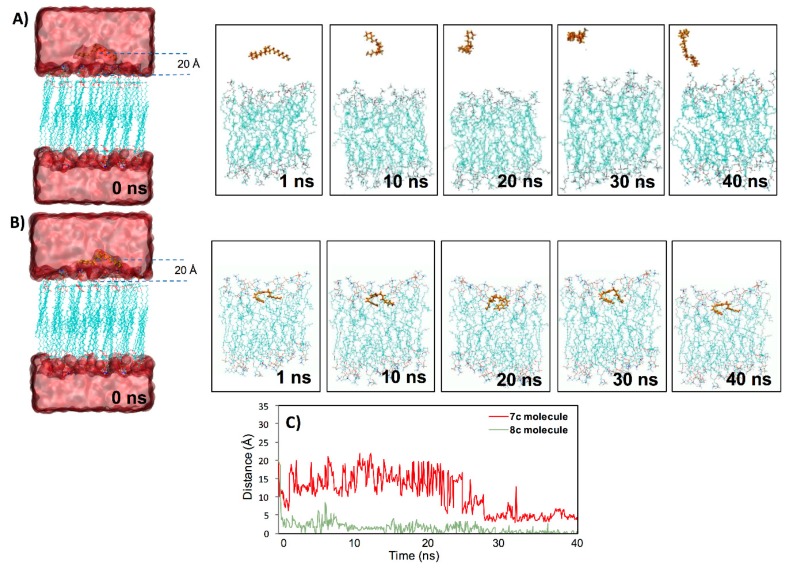
Interaction between **7c** and **8c** molecules with a lipid bilayer. Incorporation and interaction of the **7c** (**A**) and **8c** (**B**) molecules in the membrane along with 40 ns of the molecular dynamic (MD) simulation. In the figures (**A**) and (**B**) over time, the water molecules were removed to improve visualization. (**C**) Minimum distance obtained from two independent MD simulations, between the mass center of the **7c** and **8c** molecules and the surface of the lipid bilayer.

Molecular dynamic (MD) simulations for the two salts in the phosphatidyl oleoyl phosphatidylcholine (POPC) lipid bilayer were performed to study their putative incorporation into or interaction with the lipid phase. According to the antibacterial screening and biological activity determinations, **7c** and **8c** with longer aliphatic chains were expected to be incorporated into the lipid bilayer. When set at 20 Å, the distance between the ligand and the lipid center of mass immersed ligands in bulk water. The salt **7c** was highly mobile in relation to **8c**, showing a higher value of RMSD, which an average of 5.9 Å and 2.9 Å, respectively. Additionally, it was observed that the Br atom in both salts was released/separated from the molecule, which was previous to the interaction with the membrane in both MD simulations (See Videos S1 and S2). However, **7c** showed a more unstable interaction with respect to **8c**, because the interaction with the membrane was discontinuous and only occurred for a short time (Figure 2A). Meanwhile, the interaction of **8c** was constant from the first ns until the end of the MD simulation (Figure 2B and Video S2), even when it was on the surface (it was not able to completely penetrate the membrane), similar to the case of the **7c** molecule, which, in a short period of time, at the end of the MD simulation (around 3 ns during 38 to 40 ns), partially interacted with the bilayer (Figure 2A and Video S1). The incorporation of the **8c** molecule in the bilayer probably occurred via the passive diffusion phenomenon, but due to its lack of hydrophobicity (hydroxyl group of the compound), it was not incorporated into the bilayer (Figure 2B). Therefore, **8c**’s behavior was similar to that of compound [CDIM]Cl (an ionic liquid with a longer alkyl chain) previously shown by Forero-Doria et al. (2018) [24]. The authors showed, using MD simulations, how the [CDIM]Cl molecule was incorporated into and remained in the bilayer, and they related this to passive diffusion of the compound towards the membrane surface [24]. Additionally, we observed differences in the distance between the mass center of the two salts with respect to the surface of the bilayer (Figure 2C); **8c** was quickly oriented near the bilayer and for this reason, the distance was around 1 or 1.5 Å after the first nanosecond of the MD simulations. Meanwhile, **7c** showed a greater distance and even though in small moments at the final of the MD simulation it approached the bilayer, this interaction was not stable, causing the distance to be greater again (Figure 2C).

### 2.5. In Silico ADME Properties of N-alkylimidazolium Salts **7a**–**c** and **8a**–**c**

The physical properties and the ADMET parameters (adsorption, distribution, metabolism and excretion) of *N*-alkylimidazolium salts **7a**–**c** and **8a**–**c** were computed using the freely accessible web server SwissADME (http://swissadme.ch/index.php#undefined). 

The results of the in silico ADME properties of *N*-alkylimidazolium salts **7a**–**c** and **8a**–**c** are listed in Table 3, and the results suggest that there are no significant violations of Lipinski’s rule of five since all calculated physicochemical descriptors and pharmacokinetics properties are within the expected thresholds (molecular weight (g/mol) = 198–435; MLogP = 1.12–4.45; HBA = 1–3; and HBD = 0–1). The highly lipophilic character (MLogP > 4.15) of the compound **7c** can be attributed to the chain length of 10 carbon atoms, which may have made membrane penetration difficult, unlike **8c** (MlogP = 3.87), which contained a hydroxyl group in its structure; the alkyl chain of 10 carbon atoms showed a better interaction with the lipid bilayer.

## 3. Materials and Methods

### 3.1. General Information 

Reagents for synthesis were obtained from Sigma-Aldrich (St. Louis, MO, USA). ^1^H and ^13^C-NMR spectra (400 MHz for proton and 100 MHz for carbon) were recorded on an AM-400 spectrometer (Bruker, Rheinstetten, Germany). Mass spectrometry was conducted in a high-resolution mass spectrometer Exactive^TM^ Plus Orbitrap (ThermoFisher Scientific, Bremen, Germany). Scan parameters were as follows: Resolution: 140,000; AGC target: 3e6; max. inject time: 200; HESI source: sheath gas flow; 10, aux. gas flow rate: 3; sweep gas flow rate: 0; capillary temp: 250 °C; S-lens RF level: 0; heater temp: 50 °C.

### 3.2. Chemistry

#### 3.2.1. General Procedure for *N*-cinnamoylimidazole Synthesis

Briefly, a mixture of cinnamic (**1a**) or *p*-coumaric (**1b**) acid (10 mmol) and CDI (10 mmol) was subjected to grinding in a mortar grinder (FRITSCH: Pulverisette 2) at 70 rpm for 20 min, producing viscous oils as products (**3a**–**b**). The oils obtained were subject to column chromatography with DCM/MeOH 95:5 to produce a 74–92% yield. 

#### 3.2.2. General Procedure for the Synthesis of Different *N*-cinnamoylimidazolium Salts

The quaternization reaction was carried out by reacting the different *N*-cinnamoylimidazole **3a**–**b** (1 mmol) with the different alkyl bromides (hexyl (**4**), octyl (**5**), and decyl (**6**)) (1.5 mmol) under microwave radiation (250 MW) at 80 °C for 10 min and solvent-free conditions. Subsequently, the unreacted materials were removed by washing them with diethyl ether (4 × 10 mL), resulting in the crude of the reaction. Then, the residue was subject to column chromatography with Hexane/EtOAc 7:3 to produce a 40%–65% yield. 

*(E)-1-(1H-imidazol-1-yl)-3-phenylprop-2-en-1-one* (**3a**). 74% yield. ^1^H-NMR (400 MHz, MeOD): ^1^H-NMR (400 MHz, MeOD) δ 7.76 (s, 2H), 7.49–7.37 (m, 2H), 7.23 (t, *J* = 8.5 Hz, 2H), 7.02 (s, 3H), 6.39 (d, *J* = 16.0 Hz, 1H). ^13^C-NMR (100 MHz, MeOD) δ 178.69, 143.87, 136.51, 130.75, 129.95, 129.89 (2C), 129.28, 128.86 (2C), 127.75, 122.33. HRMS (HESI/Orbitrap) *m*/*z* calculated for C_12_H_10_N_2_O^+^ [M + H^+^]: 199.0866; found 199.0870.

*(E)-3-(4-hydroxyphenyl)-1-(1H-imidazol-1-yl)prop-2-en-1-one* (**3b**). 92% yield. ^1^H-NMR (400 MHz, MeOD): δ 7.66 (s, 1H), 7.19 (d, *J* = 15.9 Hz, 1H), 7.06 (d, *J* = 8.5 Hz, 2H), 6.84 (s, 2H), 6.46 (d, *J* = 8.6 Hz, 2H), 5.97 (d, *J* = 15.9 Hz, 1H). ^13^C-NMR (100 MHz, MeOD): δ 172.81, 160.72, 144.98, 136.01, 132.37, 130.77 (2C), 127.63, 122.02, 117.97, 116.75 (2C). HRMS (HESI/Orbitrap) *m*/*z* calculated for C_12_H_10_N_2_O_2_^+^ [M + H^+^]: 215.0815; found 215.0820.

*1-cinnamoyl-3-hexyl-1H-imidazol-3-ium bromide* (**7a**). 50% yield. ^1^H-NMR (400 MHz, MeOD) δ 8.01 (s, 1H), 7.63 (d, *J* = 16.0 Hz, 1H), 7.57 (d, *J* = 4.6 Hz, 2H), 7.38 (m, 3H), 7.26 (s, 1H), 7.13 (s, 1H), 6.47 (d, *J* = 16.0 Hz, 1H), 4.11–4.02 (m, 2H), 1.80 (m, 2H), 1.29 (m, 6H), 0.88 (t, *J* = 6.1 Hz, 3H). ^13^C-NMR (100 MHz, MeOD) δ 170.95, 159.13, 156.56, 145.72, 136.01, 131.24, 131.24, 129.97 (2C), 129.10 (2C), 120.17, 48.78, 32.33, 31.80, 27.13, 23.52, 14.25. HRMS (HESI/Orbitrap) *m*/*z* calculated for C_18_H_23_BrN_2_O^+^ [M^+^]: 283.1805; found 283.1820. 

*1-cinnamoyl-3-octyl-1H-imidazol-3-ium bromide* (**7b**). 65% yield. ^1^H-NMR (400 MHz, MeOD) δ 7.83 (s, 1H), 7.57 (d, *J* = 16.0 Hz, 1H), 7.53 (dd, *J* = 6.8, 1.4 Hz, 2H), 7.34 (m, 3H), 7.16 (s, 1H), 7.03 (s, 1H), 6.46 (d, *J* = 16.0 Hz, 1H), 4.01 (t, *J* = 7.1 Hz, 2H), 1.84–1.66 (m, 2H), 1.35–1.15 (m, 10H), 0.85 (t, *J* = 6.6 Hz, 3H). ^13^C-NMR (100 MHz, MeOD) δ 171.58, 144.98, 137.95, 136.20, 131.04, 129.94 (2C), 129.01 (2C), 127.52, 121.12, 121.03, 48.47, 32.87, 31.95, 30.24, 30.10, 27.49, 23.65, 14.39. HRMS (HESI/Orbitrap) *m*/*z* calculated for C_20_H_27_BrN_2_O^+^ [M^+^]: 311.2118; found 311.2130.

*1-cinnamoyl-3-decyl-1H-imidazol-3-ium bromide* (**7c**). 60% yield. ^1^H-NMR (400 MHz, MeOD) δ 7.86 (s, 1H), 7.63 (d, *J* = 16.1 Hz, 1H), 7.58 (d, *J* = 5.6 Hz, 2H), 7.39 (m, 3H), 7.21 (s, 1H), 7.07 (s, 1H), 6.49 (d, *J* = 16.0 Hz, 1H), 4.05 (t, *J*= 7.1 Hz, 2H), 1.84–1.66 (m, 2H), 1.29 (s, 14H), 0.85 (t, *J* = 6.6 Hz, 3H). ^13^C-NMR (100 MHz, MeOD) δ 171.27, 145.29, 139.38, 136.15, 131.12, 129.96 (2C), 129.05 (2C), 127.66, 120.99, 120.78, 49.29, 33.03, 31.97, 30.58 (2C), 30.39, 30.14, 27.49, 23.70, 14.41 HRMS (HESI/Orbitrap) *m*/*z* calculated for C_22_H_31_BrN_2_O^+^ [M^+^]: 339.2431; found 339.2450.

*(E)-1-(3-(4-hydroxyphenyl)acryloyl)-3-hexyl-1H-imidazol-3-ium bromide* (**8a**). 40% yield. ^1^H-NMR (400 MHz, MeOD) δ 7.63 (s, 1H), 7.56 (d, *J* = 15.9 Hz, 1H), 7.42 (m, 3H), 6.79 (m, 3H), 6.27 (d, *J* = 15.9 Hz, 1H), 4.19 (t, *J* = 7.2 Hz, 2H), 1.95–1.80 (m, 2H), 1.32 (s, 6H), 0.89 (s, 3H). ^13^C-NMR (100 MHz, MeOD) δ 171.34, 160.82, 145.85, 130.78 (2C), 127.22, 123.58, 116.60 (2C), 116.27, 50.70, 32.03, 30.83, 30.39, 26.86, 26.74, 23.29, 14.05. HRMS (HESI/Orbitrap) *m*/*z* calculated for C_18_H_23_BrN_2_O_2_^+^ [M^+^]: 299.1754; found 299.1770. 

*(E)-1-(3-(4-hydroxyphenyl)acryloyl)-3-octyl-1H-imidazol-3-ium bromide* (**8b**). 45% yield. ^1^H-NMR (400 MHz, MeOD) δ 7.52 (s, 1H), 7.47 (d, *J* = 15.9 Hz, 1H), 7.33 (m, 3H), 6.70 (m, 3H), 6.19 (d, *J* = 15.9 Hz, 1H), 4.08 (t, *J* = 7.2 Hz, 2H), 1.77 (m, 2H), 1.29–1.07 (m, 10H), 0.78 (t, *J* = 6.3 Hz, 3H). ^13^C-NMR (100 MHz, MeOD) δ 171.52, 160.96, 145.92, 130.94 (2C), 127.41, 123.73, 116.78 (2C), 50.88, 32.84, 31.82, 31.04, 30.19, 30.07, 30.00, 27.45, 27.25, 23.63, 14.39. HRMS (HESI/Orbitrap) *m*/*z* calculated for C_20_H_27_BrN_2_O_2_^+^ [M^+^]: 327.2067; found 327.2080.

*(E)-1-(3-(4-hydroxyphenyl)acryloyl)-3-decyl-1H-imidazol-3-ium bromide* (**8c**). 50% yield. ^1^H-NMR (400 MHz, MeOD) δ 7.59 (s, 1H), 7.53 (d, *J* = 15.9 Hz, 1H), 7.39 (m, 3H), 6.76 (m, 3H), 6.25 (d, *J* = 15.9 Hz, 1H), 4.15 (t, *J* = 7.2 Hz, 2H), 1.84 (dd, *J* = 13.8, 6.9 Hz, 2H), 1.42–1.14 (m, 14H), 0.84 (s, 3H). ^13^C-NMR (100 MHz, MeOD) δ 171.52, 160.99, 146.01, 130.96 (2C), 127.40, 127.04, 123.76, 116.78 (2C), 50.89, 33.01, 31.04, 30.57, 30.54, 30.39, 30.12, 30.05, 27.47, 27.25, 23.70, 14.41. HRMS (HESI/Orbitrap) *m*/*z* calculated for C_22_H_31_BrN_2_O_2_^+^ [M^+^]: 355.2380; found 355.2400.

### 3.3. Bacterial Strains

The bacterial strains were kept frozen in glycerol-lactose medium at −20 °C. The strains used were *Staphylococcus aureus* (ATCC 25923), *Staphylococcus epidermidis* (ATCC 14990), *Pseudomonas aeruginosa* (ATCC 27853), and *Acinetobacter baumannii* (ATCC 19606). Prior to the tests, they were seeded in brain heart infusion (BHI) agar and incubated for 18 h at 37 °C, after which they were spiked in a BHI broth and incubated for 18 h at 37 °C for metabolic activation of the microorganisms.

### 3.4. Determination of the Minimum Inhibitory Concentration (MIC) by Microdilution in a 96-Well Plate

The antibacterial activity of the different salts was evaluated by means of the 96-well plate microdilution assay, a technique adapted from the methodology described by Eloff et al., 1998 [35]. The bacterial inoculum was obtained from a BHI broth previously adjusted to a McFarland 0.5 and the concentration of the inoculum used for the test was a 1/10 dilution of this standard. The salts were solubilized in sterile physiological saline (0.15 M) and its concentrations were adjusted to a final well volume of 200 μL. Then, 100 μL of Mueller-Hinton broth and 100 μL of the corresponding salts solution were added to each well in duplicate. The final concentrations of each salt used were 2 mM, 1 mM, 0.5 mM, 0.25 mM, 0.125 mM, 0.0625 mM, 0.03125 mM, and 0.015625 mM. A total of 10 μL of the 0.5 McFarland bacterial suspension diluted 1/10 was added to each well. 

Bacterial growth controls (positive control) and the control of culture medium (negative control) were included in each plate, and the plates were incubated for 18 h at 37 °C in continuous agitation. Bacterial growth was revealed through the addition of 10 μL of the redox indicator 2,3,5-triphenyltetrazolium chloride (5 mg/mL) to each well. This sample was then incubated for 30 min at 37 °C under agitation, and the reddish coloration obtained was measured at an absorbance of 600 nm with a multi-plate reader. The results obtained from three independent experiments reported the lowest concentration of a salt that prevents the visible growth of the microorganism (MIC).

### 3.5. Determination of Antibiofilm Activity in 96-Well Plates

The bacterial inoculum was obtained from a BHI broth previously adjusted to a McFarland 0.5 and the concentration of the inoculum used for the test was a 1/10 dilution of this standard. The salts were solubilized in sterile physiological saline (0.15 M) and their concentrations were adjusted to a final well volume of 200 μL. Then, 100 μL of Mueller-Hinton broth and 100 μL of the corresponding salt solution were added to each well in duplicate.

A concentration corresponding to twice the MIC (MIC × 2) and 50% of the MIC (MIC × 1/2) of each salt was used for each bacterium. A total of 10 μL of the 0.5 McFarland suspension diluted 1/10 was added to each well, bacterial growth controls (positive control) and the control of culture medium (negative control) were included in each plate, and the plates were incubated for 18 h at 37 °C without agitation. 

To reveal the formation of biofilm, 20 μL of 0.1% crystal violet solution (pre-filtered through a 0.44 μm filter) was added to each well and incubated for 10 min at room temperature. Then, each well was washed with PBS buffer three times, the plates were allowed to dry for 15 min, and 100 μL of 95% ethanol was then added to each well, solubilizing the staining at room temperature. Finally, the content of each well was mixed for a short time and the absorbance was measured at a wavelength of 600 nm, in a Tecan Infinite^®^ 200 Pro plate reader (Männerdorf, Switzerland).

Due to the MIC value against *P. aeruginosa* being above the maximum concentration used, it was not included in this assay. Additionally, the salts **7b** and **8b** were used in the determination of antibiofilm activity because they showed defined MIC values (except for *P. aeruginosa*). The differences among these salts were obtained using one-way ANOVA with a Tukey post-test, and a p-value under 0.05 was considered statistically significative. 

### 3.6. Molecular Dynamic (MD) Simulations Assay

Molecular dynamic (MD) simulations used to study the interaction mechanism between **7c**, **8c**, and membranes were performed according to Forero-Doria et al., 2018 [26]. The two salts were placed in a periodically repeating box containing explicit TIP3P [36], defined as water molecules with a pre-equilibrated phosphatidyl oleoyl phosphatidylcholine (POPC) bilayer in a periodic boundary condition box (45 Å × 55 Å × 95 Å^3^). The SCHRÖDINGER suite with an OPLS v2005 force field [37] was used for the MD simulation. The POPC lipid bilayer, composed of 96 lipids (48 per monolayer), was hydrated on each side by 20 Å water slabs. Ligands were immersed in the TIP3P water by placing each molecule at 10 Å from the lipid bilayer. Systems were energy minimized (5000 steps), equilibrated for 1 ns, and simulated for 40 ns. Periodic boundary conditions were implemented in both systems. The MD simulation was performed at constant temperature (300 K) and pressure (1.01325 bar) values, with an isothermal-isobaric ensemble (NPT-Ensemble). The MD simulation was run for 40 ns, the motion equations were integrated with 2 fs, and the data were collected every 40 ps of the trajectory. Finally, the MDS was analyzed using the VMD software [38].

## 4. Conclusions

A series of novel *N*-alkylimidazolium salts functionalized with cinnamic and *p*-coumaric acid with different chain lengths were synthesized using a mechanochemistry approach. The synthesized salts showed good antibacterial activity against Gram-positive bacteria (chain length of 8 and 10 carbons), and activity that decreased against the studied Gram-negative bacteria, and a better antibacterial effect was found when an OH group was presented in the structure of the salt, as observed in the salt **8c**, which presented the best MIC value against *S. epidermidis* and *S. aureus*. However, when the antibiofilm activity was studied, no significant differences were found among the salts derived from *p*-coumaric, highlighting the salts derived from cinnamic acid, especially the inhibition of the formation of biofilm of *S. aureus*, with about 52% inhibition. The MD simulations showed that the salts derived from cinnamic acid were highly mobile compared with the salts derived from coumaric acid; nevertheless, the salts derived from *p*-coumaric acid showed a more stable interaction with the membrane, explaining, in part, the better antibacterial activity when the OH group was present. These findings, along with the MD simulations analysis, suggest that the salts derived from *p*-coumaric acid with chain lengths of 8 and 10 carbons are good alternatives as antibacterial compounds, but the salt derived from cinnamic acid with an alkyl chain of 8 carbons could be a good alternative as an antibiofilm compound against infections caused by *S. aureus.*

## Figures and Tables

**Table 1:** Minimum inhibitory concentration (MIC) of Gram-positive and Gram-negative bacteria.

	*S. aureus*	*S. epidermidis*	*A. baumannii*	*P. aeruginosa*
	μM			
**3a**	>2000	>2000	>2000	>2000
**7a**	2000	2000	2000	>2000
**7b**	250	125	250	>2000
**7c**	62.5	31.25	250	2000
**3b**	>2000	>2000	>2000	>2000
**8a**	2000	1000	2000	>2000
**8b**	62.5	31.25	1000	2000
**8c**	31.25	<15.625	500	1000

**Table 2:** Percentage (%) of inhibition of biofilm formation.

	*S. aureus*		*S. epidermidis*		*A. baumannii*	
	MIC × 2	MIC × 1/2	MIC × 2	MIC × 1/2	MIC × 2	MIC × 1/2
**7b**	52 ± 4% **	20 ± 6%	28 ± 5%	3 ± 2% ***	34 ± 4% *	1 ± 0.8% *
**8b**	34 ± 3%	33 ± 7%	35 ± 6%	33 ± 6%	20 ± 5%	20 ± 4%

* *p* < 0.05; ** *p* < 0.01, and *** *p* < 0.001 against **8b**.

**Table 3:** Physicochemical descriptor calculated by the SwissADME simulation.

Comp.	MW ^a^	MlogP ^b^	*n*HBA ^c^	*n*HBD ^d^	*n*RB ^e^	TPSA (Å^2^) ^f^
**3a**	198.22	1.70	2	0	3	34.89
**7a**	363.29	3.57	1	0	8	25.88
**7b**	391.35	4.02	1	0	10	25.88
**7c**	419.40	4.45	1	0	12	25.88
**3b**	214.22	1.12	3	1	3	55.12
**8a**	379.29	2.99	2	1	8	46.11
**8b**	407.34	3.44	2	1	10	46.11
**8c**	435.40	3.87	2	1	12	46.11

^a^ Molecular weight; ^b^ calculated lipophillicity (MLog *P*_o/w_); ^c^ number of hydrogen bond acceptors; ^d^ number of hydrogen bond donors; ^e^ number of rotatable bonds; ^f^ topological polar surface area.

## Supplementary Materials

The following are available online, Figures S1–S16 are ^1^H and ^13^C NMR spectrums, Videos S1–S2: MD simulations of compounds **7c** and **8c**, respectively.
