# Supplementary material for: N-alkylimidazolium Salts Functionalized with p-Coumaric and Cinnamic Acid: A Study of Their Antimicrobial and Antibiofilm Effects
Source: Molecules. 2019 Sep 26;24(19):3484. doi: 10.3390/molecules24193484 (PMC6803990; doi:10.3390/molecules24193484)
Supplement: Supplementary file 1 [file molecules-24-03484-s001.zip › Supporting information.docx]

**Supplementary Information**

N-alkylimidazolium salts functionalized with p-coumaric and cinnamic acid: study of its antimicrobial and antibiofilm effects

*Oscar Forero-Doria^1^, Ramiro Araya-Maturana^1,3^, Anggela Barrientos-Retamal^1^, Luis Morales-Quintana^4^, Luis Guzmán^2*^.*

^1^Instituto de Química de Recursos Naturales, Universidad de Talca, Talca, Chile; ^2^Departamento de Bioquímica Clínica e Inmunohematología, Facultad de Ciencias de la Salud, Universidad de Talca, P.O. Box 747, Talca, Chile; ^3^Programa de Investigación Asociativa en Cáncer Gástrico (PIA-CG), Universidad de Talca, Talca 3460000, Chile; ^4^Multidisciplinary Agroindustry Research Laboratory, Instituto de Ciencias Biomédicas, Universidad Autónoma de Chile, Talca, Chile.

**I. 1H and 13C NMR spectra:**

**Figure S1.** ^1^H NMR spectrum (400 MHz, MeOD) of compound **3a.**

**Figure S2.** ^13^C NMR spectrum (100 MHz, MeOD) of compound **3a.**

**Figure S3.** ^1^H NMR spectrum (400 MHz, MeOD) of compound **3b.**

**Figure S4.** ^13^C NMR spectrum (100 MHz, MeOD) of compound **3b.**

**Figure S5.** ^1^H NMR spectrum (400 MHz, MeOD) of compound **7a.**

**Figure S6.** ^13^C NMR spectrum (100 MHz, MeOD) of compound **7a.**

**Figure S7.** ^1^H NMR spectrum (400 MHz, MeOD) of compound **7b.**

**Figure S8.** ^13^C NMR spectrum (100 MHz, MeOD) of compound **7b.**

**Figure S9.** ^1^H NMR spectrum (400 MHz, MeOD) of compound **7c.**

**Figure S10.** ^13^C NMR spectrum (100 MHz, MeOD) of compound **7c.**

**Figure S11.** ^1^H NMR spectrum (400 MHz, MeOD) of compound **8a.**

**Figure S12.** ^13^C NMR spectrum (100 MHz, MeOD) of compound **8a.**

**Figure S13.** ^1^H NMR spectrum (400 MHz, MeOD) of compound **8b.**

**Figure S14.** ^13^C NMR spectrum (100 MHz, MeOD) of compound **8b.**

**Figure S15.** ^1^H NMR spectrum (400 MHz, MeOD) of compound **8c.**

**Figure S16.** ^13^C NMR spectrum (100 MHz, MeOD) of compound **8c.**
